# An adenovirus prime/plasmid boost strategy for induction of equipotent immune responses to two dengue virus serotypes

**DOI:** 10.1186/1472-6750-7-10

**Published:** 2007-02-15

**Authors:** Saima Khanam, Pilankatta Rajendra, Navin Khanna, Sathyamangalam Swaminathan

**Affiliations:** 1 RGP Group, International Centre for Genetic Engineering & Biotechnology, PO Box 10504, Aruna Asaf Ali Marg, New Delhi 110016, India

## Abstract

**Background:**

Dengue is a public health problem of global significance for which there is neither an effective antiviral therapy nor a preventive vaccine. It is a mosquito-borne viral disease, caused by dengue (DEN) viruses, which are members of the *Flaviviridae *family. There are four closely related serotypes, DEN-1, DEN-2, DEN-3 and DEN-4, each of which is capable of causing disease. As immunity to any one serotype can potentially sensitize an individual to severe disease during exposure to a heterologous serotype, the general consensus is that an effective vaccine should be tetravalent, that is, it must be capable of affording protection against all four serotypes. The current strategy of creating tetravalent vaccine formulations by mixing together four monovalent live attenuated vaccine viruses has revealed the phenomenon of viral interference leading to the manifestation of immune responses biased towards a single serotype.

**Results:**

This work stems from the emergence of (i) the DEN virus envelope (E) domain III (EDIII) as the most important region of the molecule from a vaccine perspective and (ii) the adenovirus (Ad) as a promising vaccine vector platform. We describe the construction of a recombinant, replication-defective Ad (rAd) vector encoding a chimeric antigen made of in-frame linked EDIIIs of DEN virus serotypes 2 and 4. Using this rAd vector, in conjunction with a plasmid vector encoding the same chimeric bivalent antigen, in a prime-boost strategy, we show that it is possible to elicit equipotent neutralizing and T cell responses specific to both DEN serotypes 2 and 4.

**Conclusion:**

Our data support the hypothesis that a DEN vaccine targeting more than one serotype may be based on a single DNA-based vector to circumvent viral interference. This work lays the foundation for developing a single Ad vector encoding EDIIIs of all four DEN serotypes to evoke a balanced immune response against each one of them. Thus, this work has implications for the development of safe and effective tetravalent dengue vaccines.

## Background

DEN viruses, of which there exist four distinct serotypes (DEN-1, -2, -3 and -4), belonging to the family *Flaviviridae*, are transmitted to humans by the mosquito *Aedes aegypti*. Infection with DEN viruses poses a significant public health threat to ~40% of the global population. DEN virus infections, of which ~100 million cases occur annually, produce symptoms ranging from mild fever to severe hemorrhagic, potentially fatal, fever [1-3]. The pathogenesis of severe disease is not very well understood. Though several factors such as virulence of the infecting virus, age and genetic predisposition of the patient are implicated [4,5], the most important factor is held to be sequential infection by different serotypes in areas of endemicity [1-3]. Antibodies elicited by a given DEN virus serotype, which confer lifelong homologous immunity to that serotype, can cross-react with, but not protect against the remaining serotypes. These in fact have the potential to exacerbate disease severity through antibody dependent enhancement (ADE), during secondary infection with a different serotype [6,7]. Additionally, pre-existing memory T cell populations with lower avidity for the secondary serotype may be responsible for sub-optimal viral clearance and enhanced pro-inflammatory responses [8]. Therefore, an ideal dengue vaccine should be 'tetravalent', capable of affording protection against all four DEN virus serotypes [9,10].

In an effort to develop tetravalent dengue vaccines, several investigators are currently exploring plasmid DNA-based experimental vaccines in mice [11-13] and monkeys [14]. Simultaneously, some groups are also investigating recombinant protein-based monovalent vaccine candidates [15,16]. Of the numerous DEN vaccine approaches being explored, live attenuated [17-23] and yellow fever vaccine vector YF17D-based chimeric [24-27] vaccines are in advanced stages of development [10,28]. Vector backbones based on attenuated DEN viruses are also being used to develop intertypic chimeric vaccines [29,30]. Uniformly, all these vaccine candidates are 'monovalent' in that they are specific to a single DEN virus serotype. All these strategies rely on physically mixing the four monovalent vaccines to generate empirical 'tetravalent' formulations [reviewed in [31,32]]. It is becoming increasingly apparent that these formulations have the potential to elicit a skewed immune response, predominantly to a single serotype [18,23,25]. This has been attributed to 'viral interference', a poorly understood phenomenon, associated with increased replication of one of the monovalent vaccine viruses in the tetravalent formulation [reviewed in [31,32]]. This has necessitated the testing of multiple, empirical tetravalent formulations [20,22,27,30]. Recently, Zhou and Deem have suggested that injecting the monovalent vaccines at separate sites may alleviate this problem [33].

The objective of this work is to seek proof-of-concept for the hypothesis that a single vaccine vector targeting multiple DEN virus serotypes has the potential to bypass the risk of viral interference inherent in the currently prevalent approach of mixing 'monovalent' vaccines. For this purpose, we have developed a rAd vector encoding a novel 'bivalent' antigen, based on EDIII of DEN-2 and DEN-4 viruses. The choice of EDIII was based on several reasons. First, EDIII has emerged as the most important domain from a vaccine perspective. It has been shown that the host cell surface receptor-binding motif of the E protein maps to EDIII [34]; further serotype-specific multiple neutralizing epitopes have been localized to this domain [35-37]. Importantly, EDIII has only a very low intrinsic potential for eliciting cross-reactive antibodies against heterologous DEN virus serotypes [38,39], implicated in severe pathogenesis. Second, DEN-2 virus EDIII protein (EDIII-2) can block DEN-2 virus infectivity [40,41]. Third, several groups [13,15,16,38,39] including ours [42,43] have shown that EDIII-encoding gene-based vaccine candidates and recombinant EDIII fusion proteins elicit neutralizing antibodies.

In this paper, we describe the construction and characterization of a bivalent rAd vector encoding a chimeric antigen created by linking EDIIIs of DEN virus serotypes 2 and 4 (EDIII-4/2 antigen), and show that it elicits comparable levels of virus-neutralizing antibodies and T cell responses specific to these two serotypes.

## Results

### Creation of an EDIII-based bivalent antigen-encoding adenovirus vector, rAd-Bg

**Figure 1:**
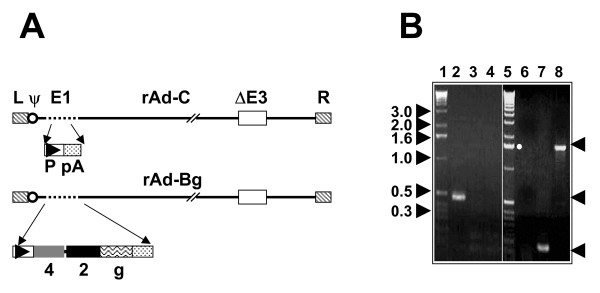
**The rAds constructed for this study**. (A) Schematic representation of the linear genomes of the rAd-C and rAd-Bg viruses. The dashed line, close to the left end represents the position of region E1. The open box located towards the right end represents a ~2.7 Kb deletion in the non-essential E3 region (ΔE3). The hatched boxes at either end represent the left (L) and right (R) inverted terminal repeats (ITRs), and the open circle close to the L-ITR represents the packaging signal (ψ). In the rAds, region E1 is replaced either by an empty expression cassette (rAd-C) or the EDIII-4/2 expression cassette (rAd-Bg). Components of the expression cassettes are indicated by the following abbreviations, P: CMV promoter (the arrow indicates its orientation); 4 and 2: DEN-4 and DEN-2 EDIII-encoding regions, respectively, of the bivalent *EDIII-4/2 *gene; g: enhanced green fluorescent protein gene (fused in-frame to the *EDIII-4/2 *gene) and pA: SV40 polyadenylation signal. (B) Characterization of the rAd genomes by PCR. The picture depicts agarose gel analysis of PCR products generated using wt Ad5 (lanes 2, 6), rAd-C (lanes 3, 7) or rAd-Bg (lanes 4, 8) viral DNA templates. Primers used were either E1-specific (lanes 2–4) or specific to insert-flanking sequences (lanes 6–8). DNA size markers were run in lanes 1 and 5. Sizes of the lane 1 markers (in Kb) are indicated to the left of the panel. The position of the 1.6 Kb band in lane 5 is indicated by a white dot for orientation. The arrows to the right indicate the positions of the amplicons corresponding to the bivalent insert (top), E1 region (middle) and the insert-less vector sequences (bottom).

Two rAd vectors, shown in Figure 1A, were constructed for this study. The test vector, rAd-Bg, carries the human cytomegalovirus (CMV) promoter-driven chimeric *EDIII-4/2 *antigen gene (derived by linking the EDIII-encoding cDNAs generated from DEN-4 and DEN-2 viral genomic RNAs) fused to a 3' green fluorescent protein gene (*GFP*) tag, inserted into the Ad5 genome, in place of its E1 region. The control vector, rAd-C, is similar in design, with the exception that it carries an empty CMV expression cassette. Aliquots of the rAd viral DNA were subjected to PCR analysis using Ad *E1*- and insert-flanking primers as described earlier [42]. The results are shown in Figure 1B. The *E1*-specific primers produced the predicted ~0.47 Kb amplicon when the template was DNA from wild type (wt) Ad (lane 2), but not when it was from either the rAd-C (lane 3), or rAd-Bg (lane 4) viruses. PCR with insert-flanking primers resulted in the amplification of a ~1.5 Kb cDNA only from rAd-Bg (lane 8) but not from the wt Ad DNA template (lane 6). From rAd-C viral DNA as the template, the insert-flanking primers amplified a ~0.1 Kb vector derived cDNA product (lane 7). Extensive restriction mapping of the rAd-C and rAd-Bg viral DNAs were found to be consistent with the construction strategy (*data not shown*).

**Figure 2:**
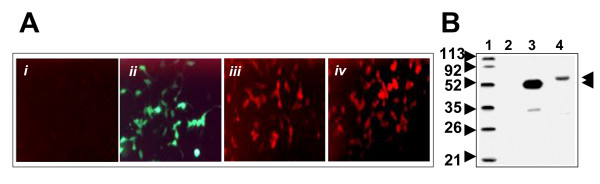
**rAd-mediated expression of rEDIII-4/2 protein as aGFP fusion**. (A) Detection of rAd-mediated bivalent antigen expression by fluorescence microscopy. HEK 293 cells were either infected with rAd-C (panel *i*), or rAd-Bg (panel *ii-iv*) for 24 hours and examined either directly for GFP fluorescence (panels *i *and *ii*) or analyzed by IFA, using either mAb 3H5 (panel *iii*) or MAB8704 (panel *iv*), as the primary antibody in conjunction with rhodamine-conjugated anti-murine IgG as the secondary antibody. (B) Immunoprecipitation of the bivalent protein from infected HEK 293 cells. Cells were infected with rAd-C (lane 2), a rAd expressing DEN-2 E (lane 3 [ref. 45]) or rAd-Bg (lane 4) viruses. Infected cells were metabolically labeled with [^35^S]-methionine, lysed, immunoprecipitated with 3H5 mAb, and analyzed on a denaturing 15% polyacrylamide gel. Pre-stained protein markers were run on the same gel (lane 1); their sizes (in kDa) are indicated on the left. The arrows on the right show the positions of the bivalent protein (upper) and the E protein (lower).

We next examined the ability of the rAd-Bg virus to express the bivalent antigen by direct fluorescence microscopy to detect its GFP tag. In the experiment shown in Figure 2A, HEK 293 cells were observed ~24 hours after infection with rAd-Bg (panel *ii*). Detection of the C-terminal GFP tag suggests successful rAd-mediated expression of the bivalent antigen. On the other hand, rAd-C virus-infected cells (panel *i*), analyzed in parallel, did not display any green fluorescence. That the rAd-C virus had efficiently infected the cells was evident from the cytopathicity observed by visible light microscopy (*data not shown*). In addition, we carried out indirect immunofluorescence assays (IFA) to detect the EDIIIs corresponding to DEN-2 and DEN-4 in the bivalent antigen. To this end, we probed rAd-Bg infected cells, separately, with monoclonal antibodies (mAbs) 3H5 and MAB8704 to identify EDIIIs corresponding to DEN-2 and DEN-4, respectively. As the bivalent antigen was GFP-tagged, we used anti-murine IgG-rhodamine conjugate as the secondary antibody in the IFA experiment. 3H5 is a DEN-2 serotype-specific mAb, which specifically recognizes an epitope that maps to amino acid (aa) residues 386–397 of the E protein [44]. As this epitope falls within the DEN-2 EDIII region, this mAb is expected to recognize the rEDIII-4/2 bivalent antigen as well. MAB 8704 is a DEN-4 virus-specific mAb. We found that it was able to specifically recognize *E. coli*-expressed DEN-4 EDIII (*our unpublished data*). Thus, this mAb also is expected to recognize the rEDIII-4/2 bivalent antigen. Consistent with this, probing rAd-Bg infected cells with mAbs 3H5 (panel *iii*) and MAB8704 (panel *iv*) resulted in red immunofluorescence. We also identified the bivalent antigen in rAd-Bg virus-infected cells using an immunoprecipitation approach (Figure 2B). In this experiment, we used mAb 3H5 to immunoprecipitate the bivalent antigen expressed in infected HEK 293 cells. Consistent with the direct fluorescence and IFA data, the 3H5 mAb specifically identified a protein of the predicted size (~55 kDa) in rAd-Bg infected (lane 4), but not in rAd-C infected (lane 2) and mock infected (*data not shown*) HEK 293 cells. As a positive control, a parallel immunoprecipitation was performed using cells infected with a previously reported rAd [45], expressing aa 1–395 of the DEN-2 virus E protein (lane 3). Taken together, these data showed that the rAd-Bg virus is capable of expressing the intended chimeric bivalent antigen in infected mammalian cells.

### The bivalent antigen-induced antibodies recognize and neutralize the infectivity of DEN-2 and DEN-4, but not DEN-1 and DEN-3 viruses

**Figure 3:**
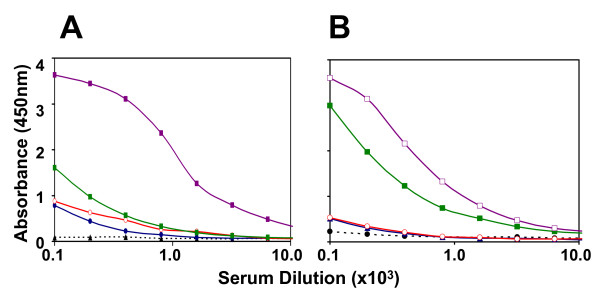
**Reactivity of immune sera towards monovalent and bivalent rEDIII proteins**. (A) Antibody titers in serial dilutions of murine sera drawn 1 week after priming with rAd-Bg (blue) and 1 week following the first (red), second (green) and final (violet) boosts with pVAX-EDIII-4/2 plasmid were determined in an ELISA using the rEDIII-4/2 bivalent protein as the capture antigen. The black dashed curve indicates ELISA reactivity of control sera, drawn from rAd-C primed/pVAX1-boosted mice after the final boost. (B) Antibody titers in serial dilutions of murine sera drawn from rAd-Bg primed/pVAX-EDIII-4/2 boosted mice, one week after the final boost. ELISA was performed using rEDIII-1 (blue), rEDIII-2 (green), rEDIII-3 (red) and rEDIII-4 (violet) proteins as capture antigens. The black dashed curve indicates ELISA reactivity of control sera using a mixture of the four rEDIII proteins as capture antigen. All ELISA values in this experiment represent the average of two separate determinations.

A major objective of this study was to investigate if a single rAd vector encoding an EDIII-based bivalent DEN antigen would elicit antibodies specific to both the constituent serotypes. To this end, we undertook mice immunization experiments. As anti-Ad antibodies resulting from the initial immunization of the mice can potentially interfere with the efficacy of the rAd vector to function as a vaccine carrier during booster immunizations, we adopted a heterologous prime/boost strategy that entailed priming the animals with the rAd vector, followed by boosting with a plasmid vector encoding the same bivalent antigen gene. Sera were collected periodically after priming and after each boost. We performed two series of ELISAs using these sera. In the first experiment, we determined antibody titers after each immunization, using the bivalent r-EDIII-4/2 protein as the capture antigen, as shown in Figure 3A. It is evident from the data presented that initial antibody titers which were relatively low, progressively increased after each boost, with maximal titers being observed after the final plasmid boost. In the next experiment, we analyzed the capacity of sera obtained after the final boost, to specifically react with monovalent EDIII proteins corresponding to each of the four DEN virus serotypes. Accordingly, in this experiment antibody titers were determined using using rEDIII-1, rEDIII-2, rEDIII-3 and rEDIII-4 proteins, as capture antigens. The results of this experiment, depicted in Figure 3B, showed that serum from the immunized animals manifested high levels of reactivity towards the rEDIII proteins corresponding to DEN virus serotypes 2 and 4, but not to serotypes 1 and 3.

**Figure 4:**
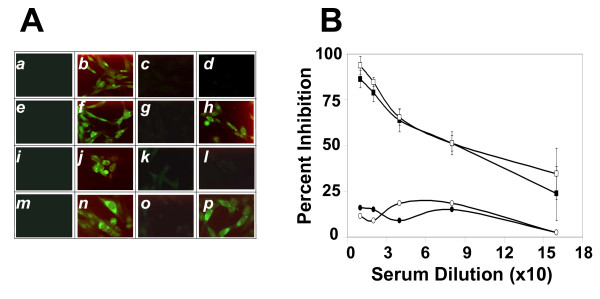
**Simultaneous induction of antibodies that recognize and neutralize DEN virus serotypes 2 and 4**. (A) IFA of DEN virus-infected cells using murine antisera. BHK cells were either mock infected (panels *a*, *e*, *i *and *m*) or infected separately with DEN-1 (panels *b*, *c*, *d*), DEN-2 (panels *f*, *g*, *h*), DEN-3 (panels *j*, *k*, *l*) and DEN-4 (panels *n*, *o*, *p*) viruses. One day after infection, cells were fixed and probed separately with different antisera as the source of primary antibodies. The antisera were obtained from mice, immunized with purified recombinant proteins [rEDIII-1 (panels *a*, *b*), rEDIII-2 (panels *e*, *f*), rEDIII-3 (panels *i*, *j*), and rEDIII-4 (panels *m*, *n*)] and rAd/plasmid [rAd-C/pVAX1 (panels *c*, *g*, *k*, *o*) or rAd-Bg/pVAX-EDIII-4/2 (panels *d*, *h*, *l*, *p*)]. The resultant virus/antibody complexes were visualized using anti-mouse IgG-FITC conjugate. (B) LLCMK2 monolayers were separately infected with each of the four DEN viruses (DEN-1: filled circles; DEN-2: filled squares; DEN-3: open circles; & DEN-4: open squares) that had been pre-incubated with serial two-fold dilutions of pooled immune serum drawn from the rAd-Bg primed/plasmid pVAX-EDIII-4/2 boosted animals. The resultant plaque counts, expressed as percent inhibition of virus infectivity (with reference to the number of plaques generated in the absence of antiserum, which was taken to represent 100% infectivity) are plotted as a function of antiserum dilution. Each data point shown represents the mean of four replicate assays (the error bars represent SD).

To address the question if these antibodies would display this serotype preference toward the DEN viruses themselves, we carried out an immunofluorescence-based assay. BHK cells were separately infected with each of the four DEN virus serotypes and probed with the test antiserum (obtained from rAd-Bg primed/bivalent plasmid boosted animals) in conjunction with a secondary antibody-FITC conjugate. The prediction was that the test antiserum should light up cells infected with DEN-2 and DEN-4, but not DEN-1 and DEN-3 viruses. The results of this experiment which substantiate this are presented in Figure 4A. Panels in the first (*a, e, i *and *m*) and second (*b, f, j *and *n*) columns depict the negative and positive controls, respectively. DEN-1 (panel *b*), DEN-2 (panel *f*), DEN-3 (panel *j*) and DEN-4 (panel *n*) viruses were detected using murine polyclonal antibodies raised against *E. coli*-expressed rEDIII-1, rEDIII-2, rEDIII-3 and rEDIII-4 proteins, respectively. The detection of immunofluorescence in panels *b*, *f*, *j *and *n *demonstrates that DEN-1, 2, 3 and 4 viruses, respectively, had successfully infected the cells in this experiment. Mock-infected cells did not fluoresce with any of these antibodies (panels *a, e, i *and *m*), indicating that the antibodies used are virus-specific. The control serum (obtained from rAd-C primed/empty plasmid boosted animals) also resulted in no fluorescence in DEN virus-infected cells (panels *c, g, k *and *o*). This suggests that the vaccine vectors *per se *do not induce a DEN virus-specific immune response. Consistent with the ELISA data, the use of test serum as the source of primary antibodies in the assay resulted in the identification of DEN-2 (panel *h*) and DEN-4 (panel *p*), but not DEN-1 (panel *d*) and DEN-3 (panel *l*) viruses, as predicted. Taken together, the data strongly suggest that the bivalent EDIII-4/2 antigen elicits antibodies that specifically recognize DEN-2 and DEN-4 viruses with little evidence of cross-reactivity towards DEN-1 and DEN-3 viruses.

While the data obtained so far indicated that the EDIII-4/2 antigen induces a bivalent response targeting DEN-2 and DEN-4 viruses, it does not provide any information regarding the capacity of these antibodies to neutralize their infectivity. To this end, we performed plaque reduction neutralization tests (PRNTs), by plaqueing the antibody-incubated DEN viruses on LLCMK2 cells. Figure 4B presents the percent reduction in virus infectivity as a function of test serum dilution for each of the four DEN virus serotypes. The data show that the test serum displayed PRNT_50 _titers of ~1:80 towards both DEN-2 and DEN-4 viruses, with no significant neutralizing antibody titers towards DEN-1 and DEN-3 viruses.

### Induction of DEN-2 and DEN-4 virus-specific T cell responses

**Figure 5:**
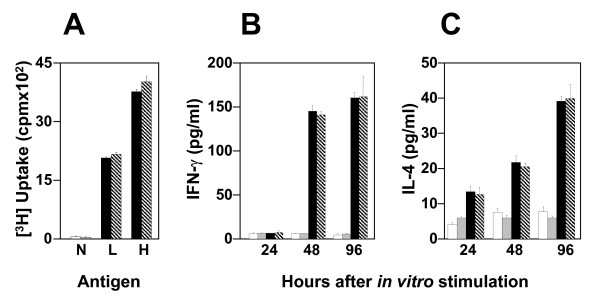
**Analysis of T cell responses elicited by the bivalent antigen**. Splenocytes were obtained from mock-immunized (open bars) and test-immunized (gray, black and hatched bars) animals, 7 weeks after the final immunization and placed in culture. They were either mock-stimulated (open and gray bars) or stimulated *in vitro*, either with DEN-2 (black bars) or DEN-4 (hatched bars) for 96 hours for performing the T cell assays. Tritiated [^3^H] thymidine uptake was determined in a scintillation counter (A). Aliquots of the culture supernatant withdrawn at the indicated time points were assayed for the presence of IFN-γ (B) and IL-4 (C) by solid phase ELISA. Data depicted represent the mean value of three separate determinations (the error bars represent SD). In panel A, two dosage levels of virus were used for *in vitro *stimulation [L, low (0.015 PFU/cell); and H, high (0.03 PFU/cell); N, no antigen]. Data in panels B and C were generated using splenocytes stimulated with the high dose.

Spleen cells in culture, derived from control and test mice, were stimulated *in vitro *with DEN-2 and DEN-4 viruses, separately, and monitored for proliferation responses and cytokine (IFN-γ and IL-4) production, as depicted in Figure 5. Splenocytes from test mice manifested significant and similar levels of proliferation in response to *in vitro *stimulation with both DEN-2 and DEN-4 viruses, compared to mock-stimulated splenocytes (Figure 5A). Control splenocytes did not manifest any proliferative response regardless of whether they were stimulated or not. The proliferative response of the test splenocytes was accompanied by IFN-γ secretion (Figure 5B). The elevated levels of IFN-γ, first evident at 48 hours post stimulation, persisted for the duration of the experiment. Again, as seen in the thymidine uptake experiment, both DEN-2 and DEN-4 virus stimulation resulted in comparable levels of IFN-γ production. At all time points tested, mock-stimulated test cells did not secrete discernible levels of IFN-γ. When control mouse spleen cell cultures were tested, IFN-γ was barely detectable in mock- and virus-stimulated culture supernatants. With regard to IL-4, the splenocytes manifested an increase in the levels of IL-4 secretion in response to virus stimulation, but it was not dramatic (as seen for IFN-γ), as basal IL-4 levels in control splenocytes were somewhat elevated. The magnitude of stimulation of IL-4 secretion in test splenocytes with respect to mock-stimulated cells, which was about 2 fold higher at 24 hours, increased to about 5 fold at 96 hours (Figure 5C). Taken together, these experiments demonstrate that the bivalent antigen, elicits DEN-2 and DEN-4 virus-specific T-cell responses. Interestingly, the magnitude of these responses was more or less equivalent for the two serotypes.

## Discussion

This work was driven by the hypothesis that switching from a strategy reliant on mixing four monovalent attenuated RNA viruses to a single tetravalent DNA viral vaccine vector may provide a means of circumventing viral interference and the associated risk of ADE. In this regard, Ad, a DNA virus, is emerging as a promising vaccine platform [46,47]. Recent data on the use of rAd vectors to develop experimental HIV-1 vaccines are encouraging. As a first step towards our objective, we recently showed that a rAd vector harboring the DEN-2 E gene could elicit DEN-2 virus-neutralizing antibodies in mice [45]. Subsequently, using EDIII of DEN-2 virus, in the context of the rAd vector system, we showed that it was able to induce DEN-2 virus-specific neutralizing antibodies and T cell responses as effectively as the complete E protein [42]. In the current study, we have created and analyzed a bivalent rAd vector. The purpose of this work was to obtain proof for the hypothesis that a single rAd-based dengue vaccine vector could elicit a balanced immune response to more than one DEN virus serotype.

We inserted a chimeric, EDIII-based bivalent gene, *EDIII-4/2*, designed by splicing together cDNAs encoding EDIIIs of DEN-2 and DEN-4 viruses [43], in place of the E1 region of the Ad5 genome to create a bivalent rAd vector. Mice immunized with a single dose of bivalent rAd manifested modest serum titers of antibodies to DEN-2 and DEN-4 viruses. In order to avoid the possibility that anti-Ad antibodies generated after rAd priming may compromise the vector's efficacy during repeat inoculations, we boosted the rAd primed mice with a plasmid encoding the bivalent antigen. DEN virus-specific antibodies in the sera of immunized animals were assessed by several criteria. This showed that these sera contained antibodies that specifically recognized EDIII-2 and EDIII-4, but manifested barely discernible reactivity towards EDIII-1 and EDIII-3. The critical question that we addressed at this point was, do these antibodies possess the capacity to neutralize, specifically, the infectivity of DEN-2 and DEN-4 viruses? Using the rAd vector prime/plasmid boost strategy, DEN-2 and DEN-4 virus-specific PRNT_50 _titers in the sera of immunized mice were each ~1:80. Using monkeys, which do not manifest dengue disease, protective efficacy is measured in terms of reduction in viremia in animals challenged with DEN virus. In the monkey model, it has been reported that PRNT_50 _titers in the range of 1:20 to 1:80, in animals immunized with either pox virus vector based-[48] or yellow fever virus-based [27] experimental dengue vaccines, confer protection against live DEN-2 virus challenge. In the mouse model, again which does not manifest any dengue disease, protective efficacy is measured in terms of survival after lethal DEN virus challenge. As the volume of serum that one can obtain from mice is quite small, PRNT assays are usually done using pooled sera. Consequently, the precise neutralizing antibody titers in individual animals prior to challenge is not known. Thus, the relationship between neutralizing antibody titers and protective efficacy has not been clearly delineated in murine systems. However, in the absence of a good animal model for dengue, neutralizing antibody titers, determined by PRNT titers are widely accepted as surrogate markers of protective immunity. PRNT_50 _titers = 1:10 are considered as indicative of protective immunity [49,50].

Consistent with the design of the bivalent antigen, the neutralizing antibodies it elicited specifically blocked the infectivity of DEN-2 and DEN-4, but not that of DEN-1 and DEN-3 viruses. This essentially corroborates our previous findings using a bacterially expressed recombinant EDIII-4/2 bivalent antigen [43]. Further, the observed immunogenicity of the EDIII-4/2 bivalent antigen either without [43] or with the GFP tag (this study) suggests that fusion of the reporter has not adversely affected its potential to elicit DEN-2 and DEN-4 virus-specific neutralizing antibodies. The *E. coli*-expressed antigen which is insoluble needs to be empirically re-folded subjecting antigen preparations to variability. Further, protein antigens are not efficient at inducing cell-based immunity (CMI). Given that the structural integrity of EDIII is essential for its immunogenicity, the gene-based vaccines (this study), which eliminate this variability are far more desirable, with the added advantage that these can also provoke potent CMI.

Our previous [42,43] and current work suggests that EDIII is serotype-specific and that it must be possible to evoke a tetravalent response using a chimeric antigen containing EDIIIs of all four DEN virus serotypes linked to each other. While most recombinant approaches to dengue vaccines deal primarily with monovalent vaccines, recently a couple of tetravalent plasmid vaccine candidates, both based on the full-length E protein genes, have been reported. One is based on mixing four monovalent plasmid vaccines [12], and the other describes a plasmid encoding a single chimeric E gene generated by DNA shuffling [11,14]. While both these are reported to elicit neutralizing antibodies, it is difficult to correlate our data on DEN-2 and DEN-4 PRNT titers with these studies as they have employed homologous plasmid prime/plasmid boost immunizations aside from differences in immunization schedules and neutralization assay protocols. Experiments are underway to investigate the role of the DEN-2 and DEN-4 virus-specific antibodies induced by rAd-Bg immunization in affecting the replication of DEN-1 and DEN-3 viruses.

## Conclusion

Our data provide support for the hypothesis that a single vector system encoding a bivalent EDIII-based antigen has the capacity to induce a balanced immune response against both parental serotypes, thereby circumventing any potential possibility of immune interference. Thus, our data suggest that one can envisage the creation of a single antigen gene encoding EDIIIs of all four DEN virus serotypes, which could be incorporated into a single rAd vector to elicit a balanced tetravalent immune response. From a technical standpoint, it would be well within the capacity of the currently used first generation rAd vectors to accommodate the EDIIIs, but not the Es, of all four DEN virus serotypes. Prior immunity to Ad5, beyond a certain threshold, may compromise the efficacy of the Ad5 vector. Nevertheless, it has served, as a model to test our hypothesis, and it should be possible to switch to a vector with low/no seroprevalence such as Ad35, chimp Ad or adeno-associated virus in future.

## Methods

### Plasmids, viruses and cells

Plasmids pVAX1 was from Invitrogen. Plasmids pShuttle-CMV and pAdEasy-1, designed for rAd vector construction, have been described previously [51]. DEN viruses DEN-1 (Nauru Island), DEN-2 (New Guinea C), DEN-2 (H87) and DEN-4 (H241) were gifted by Dr. A. Falconar (University of Oxford, Oxford, UK). Ad5 transformed HEK 293 cell line, Baby Hamster Kidney (BHK 21) cell line, the monkey kidney cell line LLCMK2, the mosquito cell line C6/36, and the 3H5 mAb-producing hybridoma cell line, HB46, were from American Type Culture Collection, Virginia, USA, and were propagated as described earlier [42].

### Preparation of DEN virus stocks

DEN virus stocks were prepared by infecting C6/36 cells at low multiplicity for two weeks with media replacement at one week. The resultant infected cell culture supernatant was used as the source of DEN viruses. Viral titers were determined by plaque assay on LLCMK2 cells [42,43].

### Construction of rAd-C and rAd-Bg viruses

The design and construction of the bivalent gene, *EDIII-4/2*, have been described previously [43]. This gene was cloned into plasmid pVAX1 and verified to encode the predicted ~28 kDa bivalent protein in a rabbit reticulocyte expression system (T_N_T system from Promega). The 3' end of this chimeric gene was fused in-frame with the 5' end of the *GFP *gene and cloned into the Ad shuttle plasmid pShuttle-CMV. The EDIII-4/2-GFP expression cassette was then inserted into the E1 region of the Ad5 genome of plasmid pAdEasy-1 by *in vivo *recombination in *E. coli *BJ5183 [51]. The resultant rAd plasmid was digested with *Pac *I to eliminate plasmid sequences and transfected into HEK 293 cells to rescue the rAd-Bg virus. The rAd-C virus was constructed in parallel using a similar strategy except that empty pShuttle-CMV plasmid was used during *in vivo *recombination in *E. coli*. Recombinant viruses were amplified, purified on CsCl gradients, dialyzed against 1× PBS containing 10% glycerol, sterilized using syringe filters and titrated on HEK 293 cells. Both viruses yielded fairly comparable titers, within 3–4 folds of each other.

### Detection of transgene expression in rAd-Bg virus-infected cells

HEK 293 cells were infected at ~25 PFU/cell, for ~24 hours. The expression of the GFP tag was detected by direct fluorescence microscopy. The DEN-2 and DEN-4 EDIII components of the bivalent antigen were detected by IFA, using mAb 3H5 (DEN-2 virus-specific) and MAB8704 (DEN-4 virus-specific), in conjunction with anti-murine IgG-rhodamine conjugate [42]. Expression of the transgene was also detected in infected, [^35^S]-radiolabeled cells by immunoprecipitation, using the 3H5 mAb as described previously [42].

### Mouse immunization

Balb/c mice (4–6 weeks old; 5 animals per group) were immunized using a rAd prime/plasmid boost regimen. The control group was injected intraperitoneally (i.p) with 10^8 ^PFU of rAd-C vector (in 200 μl of 1× PBS), followed 15 days later by three intradermal (i.d) doses, again 15 days apart, of 50 μg plasmid pVAX1 vector (in 200 μl of 1× PBS). A similar regimen was followed for the test group, which was primed i.p. with 10^8 ^PFU of rAd-Bg vector and boosted i.d. with 50 μg plasmid pVAX-EDIII-4/2 vector. Blood was drawn 1 week after priming and after each boost for seroanalysis. Animal experiments were performed after approval of the Institutional Animal Ethics Committee.

### Seroanalysis

Antibody titers in sera of immunized mice were determined by ELISA, in 96-well plates, using monovalent and bivalent r-EDIII proteins or DEN viruses as capture antigens in conjunction with anti-murine IgG-HRPO secondary antibody conjugate/3, 3' 5, 5' tetramethyl benzidine substrate, as previously described [43]. The presence of anti-DEN-2 and anti-DEN-4 virus antibodies in immune sera (diluted 1:100) was also detected by IFA of DEN-2 and DEN-4 virus-infected BHK cells (0.1 PFU/cell) at 24 hours post-infection. Virus-bound antibodies were detected using anti-mouse IgG-FITC conjugate as done earlier [43]. Neutralizing antibody titers were determined by standard PRNT assay using LLCMK2 cells as before [42,43]. The antiserum dilution resulting in 50% reduction in plaque count (with reference to the number of plaques generated by the virus in the absence of antiserum), was expressed as the PRNT_50 _titer.

### T cell assays

Spleens were harvested from mice ~7 weeks after the last immunization and cultured in 96-well plates for monitoring proliferative response and cytokine secretion in response to *in vitro *stimulation with DEN-2 and DEN-4 viruses (each at 0.03 PFU/cell). Cells were pulsed with [^3^H]-thymidine overnight at the end of 96 hours, and proliferation was quantitated by measuring the uptake of the radioisotope in a scintillation counter. IFN-γ and IL-4 were determined in splenocyte culture supernatants, at the indicated time points, using murine Quantikine cytokine ELISA kits (R & D systems), as described previously [42,45]. All assays for T cell responses were run in triplicate and values expressed as mean ± standard deviation (SD).

## Authors' contributions

SK and PR carried out the experiments. NK participated in work coordination and provided critical inputs during manuscript preparation. SS conceived of the study, and helped in its design and execution, and drafted the manuscript. All authors read and approved the final manuscript.
